# A Star in the Brainstem Reveals the First Step of Cortical Magnification

**DOI:** 10.1371/journal.pone.0022406

**Published:** 2011-07-19

**Authors:** Kenneth C. Catania, Duncan B. Leitch, Danielle Gauthier

**Affiliations:** Department of Biological Sciences, Vanderbilt University, Nashville, Tennessee, United States of America; Georgia State University, United States of America

## Abstract

A fundamental question in the neurosciences is how central nervous system (CNS) space is allocated to different sensory inputs. Yet it is difficult to measure innervation density and corresponding representational areas in the CNS of most species. These measurements can be made in star-nosed moles (*Condylura cristata*) because the cortical representation of nasal rays is visible in flattened sections and afferents from each ray can be counted. Here we used electrophysiological recordings combined with sections of the brainstem to identify a large, visible star representation in the principal sensory nucleus (PrV). PrV was greatly expanded and bulged out of the brainstem rostrally to partially invade the trigeminal nerve. The star representation was a distinct PrV subnucleus containing 11 modules, each representing one of the nasal rays. The 11 PrV ray representations were reconstructed to obtain volumes and the largest module corresponded to ray 11, the mole's tactile fovea. These measures were compared to fiber counts and primary cortical areas from a previous investigation. PrV ray volumes were closely correlated with the number of afferents from each ray, but afferents from the behaviorally most important, 11^th^ ray were preferentially over-represented. This over-representation at the brainstem level was much less than at the cortical level. Our results indicate that PrV provides the first step in magnifying CNS representations of important afferents, but additional magnification occurs at higher levels. The early development of the 11^th^, foveal appendage could provide a mechanism for the most important afferents to capture the most CNS space.

## Introduction

The preferential allocation of cortical territory to behaviorally important sensory receptors is a hallmark feature of the mammalian brain. Every student of the neurosciences is familiar with the Penfield “homunculus” illustrating the greatly expanded representation of the hands and lips of humans relative to larger but less important sensory surfaces such as the back and legs [1] – so called “cortical magnification” [2]–[3]. What determines how much cortical territory is allocated to a sensory surface? Is cortical magnification just a reflection of subcortical maps in the thalamus and brainstem, which are in turn reflecting the density of inputs from the sensory surface?

A number of studies have attempted to address this question, sometimes with different results. This is in part because of the difficulty of accurately measuring innervation density and corresponding representational areas in the central nervous system of most species. Mice provide a favorable model system for making such measurements because each whisker on the face is represented in primary somatosensory cortex (S1) by a circular unit of cells, or “barrel” that can be easily measured [4]. Welker and Van der Loos [5] examined the relationship between innervation density of each whisker and the size of each cortical barrel in S1, convincingly demonstrating a direct, proportional relationship. Thus cortical magnification for mouse whiskers is a reflection of peripheral innervation density. The same question has sometimes been a contentious issue in the case of the primate visual system. A number of studies suggested that cortical magnification of the retinal fovea was a direct reflection of the number of corresponding ganglion cells providing output from the fovea [6]–[8]. Other investigators reported that the foveal ganglion cells were preferentially magnified in primary visual cortex [9]–[12]. Azzopardi and Cowey [13] seem to have ultimately resolved this issue finding that ganglion cells from the retinal fovea were allocated 3–6 times more cortical territory than ganglion cells from more peripheral retinal areas. But the history of different results from different investigators attempting to address this question is a testament to the difficulty in making such measurements.

Star-nosed moles have many features that make them a useful system for examining the relationship between behavior, innervation density, and central maps in the somatosensory system. The mole's star consists of 11 mechanosensory appendages, or “rays” that ring each nostril. A single central pair of rays (the 11^th^ pair) act as a tactile fovea and are used to explore objects of interest much like a retinal fovea [14]. Thus there is a substantial difference in the behavioral importance of different rays. Each ray is supplied by a large branch of the infraorbital nerve containing myelinated fibers that can be readily counted. At the level of the neocortex, the primary somatosensory area contains a series of stripes that reveal the representations of the nasal rays [15], much like cortical barrels represent whiskers [4], [16]. These features facilitated a previous investigation comparing innervation density to cortical representational size for each ray [17]. The results revealed preferential magnification of the 11^th^, foveal ray and surrounding parts of the star, mirroring the results for the primate visual system [13]. Thus in the star-nosed mole's somatosensory system, cortical magnification is not simply a reflection of innervation density, rather, the most important afferents are allocated more cortical territory. The finding raised a basic question: where does the preferential allocation of neural tissue to important afferents first occur in the somatosensory system?

Here we begin to address this question by examining the principal trigeminal sensory nucleus (PrV) in the brainstem of star-nosed moles. PrV receives afferents from the trigeminal nerve and projects (via the thalamus) to primary somatosensory cortex [18], [19]. Our results reveal a number of striking anatomical findings. First, PrV in star-nosed moles appears massively expanded compared to PrV in rodents or moles without a star. Second, the bulk of PrV is made up of a histologically distinct subnucleus containing 11 large, wedge-shaped modules that represent the 11 rays on the nose. Finally, the volumes of each ray representation in PrV do not mirror the sizes of cortical areas representing rays, indicating that neither peripheral innervation density [17], nor PrV representational volumes, directly dictate the size of cortical representations in S1. Nevertheless, important afferents are preferentially magnified in PrV.

## Materials and Methods

We examined the trigeminal nuclei from 8 star-nosed moles and one hairy-tailed mole collected in Northern Pennsylvania under permit # COL00087. Star-nosed moles were anesthetized with 15% urethane (1.0 g/kg) and 10% (10 mg/ml) ketamine (10 mg/kg) with additional supplements as needed. A small craniotomy was made over the caudal neocortex, starting 1 mm lateral to the midline, and centered roughly 2.5 mm from the midline in the medio-lateral direction, up to a prominent suture in the rostral direction, and just rostral to the cerebellum in the caudal direction. The angle on the micromanipulator was adjusted to rotate the tip of the electrode 15 degrees rostrally and recordings began with the electrode roughly 2.5 mm from the midline. With this arrangement, recordings from PrV were typically at a depth of 5,000 to 6,500 microns with responses from ray 11 found most superficially and ray 1 at the greatest depth (responses from the superior colliculus representation of the rays were sometimes encountered at 3,000–4,000 microns). Recordings were made with low-impedance tungsten electrodes (1.0–1.5 M ohms at 1000 Hz) and responses were amplified and monitored with a Powerlab 4/30 data acquisition unit using LabChart software (ADinstruments). Receptive fields for PrV were mapped onto a schematic of the star and selected penetrations were lesioned at 10 microamps for 5–10 seconds. After recording sessions, moles were given an injection of sodium pentobarbital (100 mg/kg) and perfused with phosphate buffered saline followed by 4% paraformaldehyde (one hairy-tailed mole was perfused as above, with no recordings). The brainstem was removed and cryoprotected in 30% sucrose. A number of brainstems were cut in the horizontal plane (relative to the bottom of the brainstem) and processed as described below. To obtain favorable sections of the star in PrV, several brainstems were hemisected, each half was mounted on an insect pin at the spinal cord, and oriented as follows. The ventral surface of the brainstem was used as a reference for the horizontal plane. The brainstem was inverted and held horizontally relative to the flat surface of ice on the microtome stage. As viewed from the back (caudal perspective) the back of the inverted brainstem was rotated up 45 degrees from the horizontal plane. Left brainstems were then rotated roughly 45 degrees clockwise and right brainstems were rotated roughly 45 degrees counterclockwise. Brainstems were then frozen to the block, secured with additional 30% sucrose, and sectioned. Tissue was processed for cytochrome oxidase [20] mounted on glass slides and coverslipped.

Sections were photographed with a Zeiss AxioCam HRc digital camera (Zeiss, Jena, Germany) mounted onto a Zeiss Axioskop microscope using Zeiss Axiovision 4.5 software (Carl Zeiss Microimaging, Thornwood, NY, USA). Photos of serial sections from 4 star-nosed mole brainstem cases were converted to grayscale images and imported into *Reconstruct*, version 1.1.0 [21]. Sections were aligned using blood vessels and other histological landmarks as corresponding points designated with the stamp tool, and then aligned with the rigid alignment command. Alignment was checked with the blend and flicker commands. The borders of ray modules were drawn using the trace function. *Reconstruct* calculated the final volumes of traced objects based on scale bar calibration and section thickness using the Cavalieri formula.

Our current findings at the level of PrV were compared with similar results from a previous investigation of afferent numbers and S1 cortical representational area. In the previous investigation, sensory organ number (Eimer's organs), myelinated afferent counts, and S1 cortical area representation for each ray from 4 moles was quantified and compared [17]. We used the afferent counts and cortical areas from the previous investigation for our comparisons of trigeminal volume to afferent number and cortex because it is not usually possible to make all of these measurements in the same animals (not every brainstem, cortex, and sectioned nerve produces quantifiable sections). All procedures conformed to the National Institutes of Health standards concerning the use and welfare of experimental animals and were approved by the Vanderbilt University Animal Care and Use Committee (Animal Welfare Assurance Number A-3227-01).

## Results

**Figure 1:**
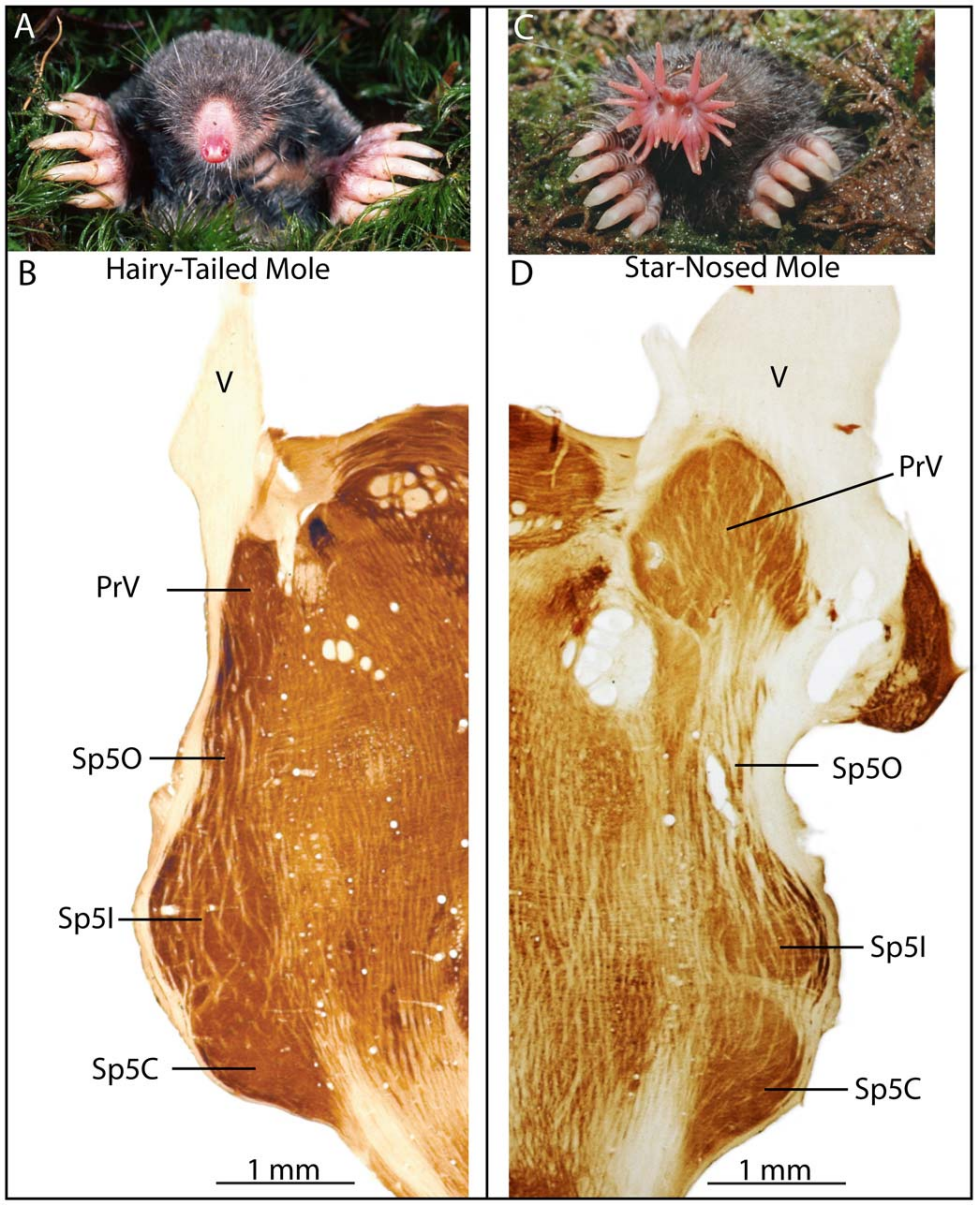
Comparison of the facial anatomy and trigeminal sensory complex of a hairy-tailed mole (*Parascalops breweri*) and star-nosed mole (*Condylura cristata*). A. A hairy-tailed mole has the typical body plan for a mole, with large forelimbs, small eyes, and a prominent but unspecialized nose. B. A horizontal section through the brainstem of a hairy tailed mole showing the trigeminal nerve (V), principal trigeminal sensory nucleus (PrV), and the spinal trigeminal nuclei (Sp50 – oral subnucleus, Sp5I - interpolar subnucleus, Sp5C - caudal subnucleus). The brainstem trigeminal nuclei of the hairy-tailed mole are similar to those of laboratory mice and rats. C. The star-nosed mole with an elaborate, mechanosensory nose. D. A horizontal section through the brainstem of a star-nosed mole. In star-nosed moles, PrV is greatly expanded in both rostral and medial-lateral directions. Note that the sections in B and D were cut horizontally relative to the separated brainstem, such that the ventral surface defined the horizontal plane.

Before discussing the details of the star representation in PrV, several unique features of the trigeminal system of star-nosed moles warrant description. These features are best appreciated by comparing the trigeminal system of the star-nosed mole to that of a more typical small mammal. Figure 1 shows horizontal brainstem sections from a star-nosed mole compared to sections from the hairy-tailed mole, processed for the metabolic enzyme cytochrome oxidase (CO). This comparison was chosen because hairy-tailed moles and star-nosed moles are close relatives that are similar in most ways, with the clear exception of the star. In horizontal sections of the hairy-tailed mole brainstem the rostral-most principal nucleus appeared as a comparatively small, thin structure just caudal to the ingress of the trigeminal nerve (Figure 1 A–B) – similar to the configuration seen in rats and mice [22]–[27].

**Figure 2:**
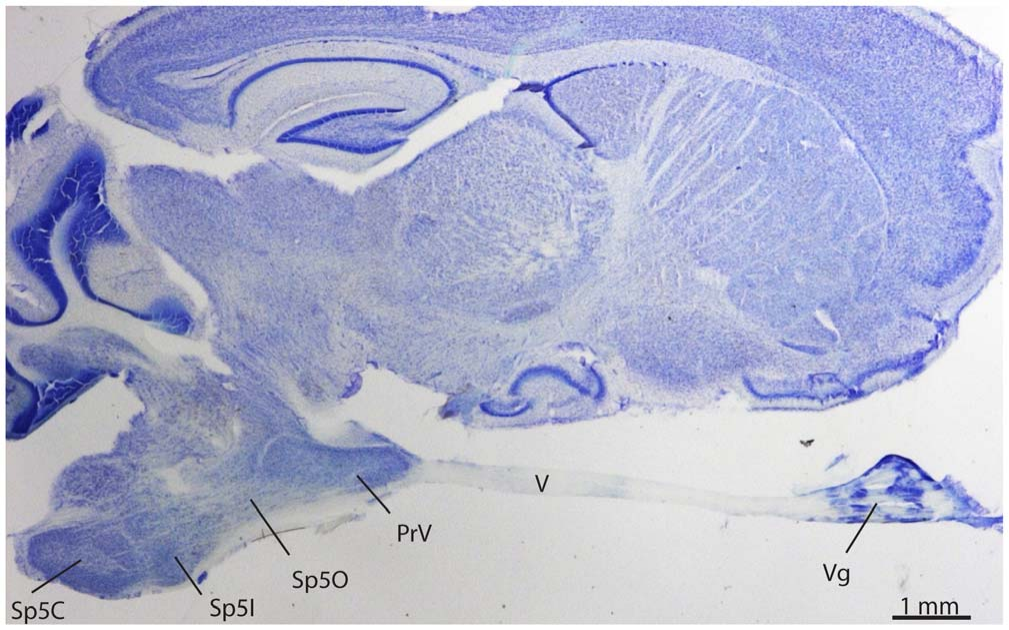
A parasagittal section of the star-nosed mole brain showing the relative size and location of the trigeminal nuclei, cranial nerve 5 (V) and the trigeminal ganglion (Vg).

In contrast, the principal nucleus of the star-nosed mole (Figure 1 C, D) was greatly enlarged, oval in shape, and extended far rostrally to partially invade the trigeminal nerve (V). This rostral expansion was so great that in some coronal sections portions of the trigeminal nerve and PrV appeared separate from the rest of the brainstem (not shown). Figure 2 shows the extent of the star-nosed mole trigeminal complex in a parasagittal, nissl stained section, further illustrating this rostral expansion and the relationship of the trigeminal ganglion to PrV. In addition to its large size (see next section for volumes), PrV was subdivided into a series of modules separated by light septa. These subdivisions seemed similar to the stripes that represent the star in the mole's somatosensory cortex [15]. This impression was strengthened when reconstruction of horizontal sections revealed a total of 11 stripes, corresponding to the number of nasal rays on each side of the star. To further investigate the relationship between the brainstem stripes and the star, multiunit electrophysiological recordings were made from PrV. At the same time, we experimented with different angles for sectioning the brainstem that allowed for the best visualization of the modules in single sections.

### A Star in PrV

**Figure 3:**
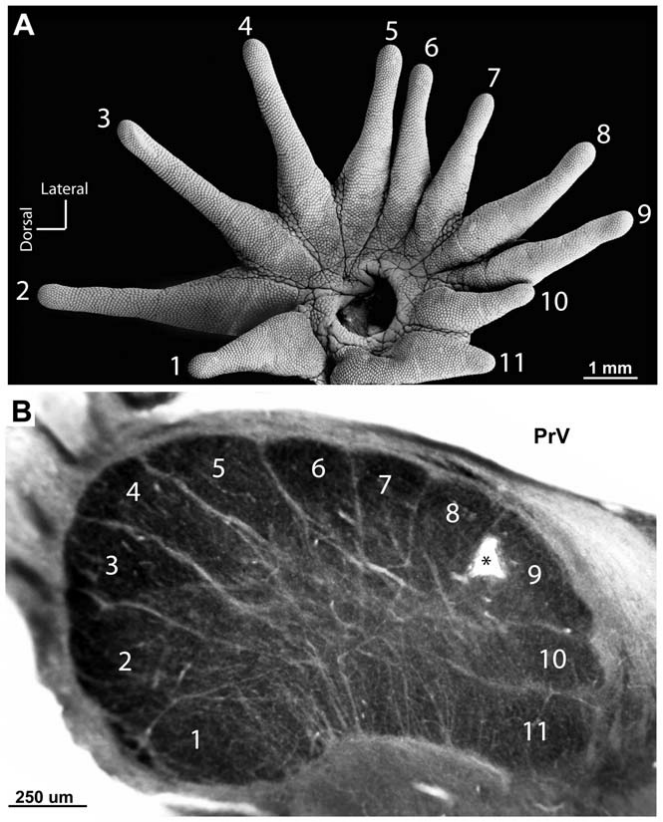
A star pattern visible in PrV. A. Half of the star rotated 90 degrees counterclockwise showing the 11 rays that ring the nostril. The relatively small 11^th^ ray acts as the tactile fovea. B. When the brainstem is properly oriented (see materials and methods) sections reveal 11 distinct modules in PrV.

**Figure 4:**
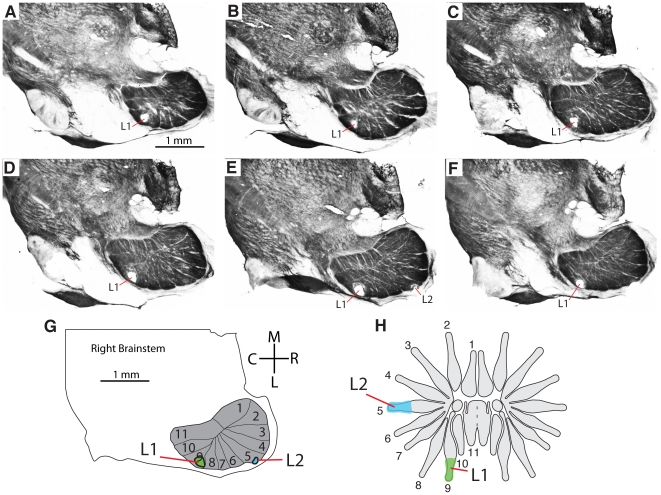
Microelectrode recordings from PrV related to brainstem anatomy. A–F. A series of sections through the brainstem showing the representation of the star in PrV and the locations of 2 microlesions made during the recordings (L1, L2). G. Drawing of the brainstem showing the star subnucleus in PrV and the locations of the microlesions made during recordings. H. Receptive fields for neurons responding at lesioned sites L1 and L2. Axes are approximate in “G” as the brainstem was rotated to obtain favorable sections (see materials and methods).

**Figure 5:**
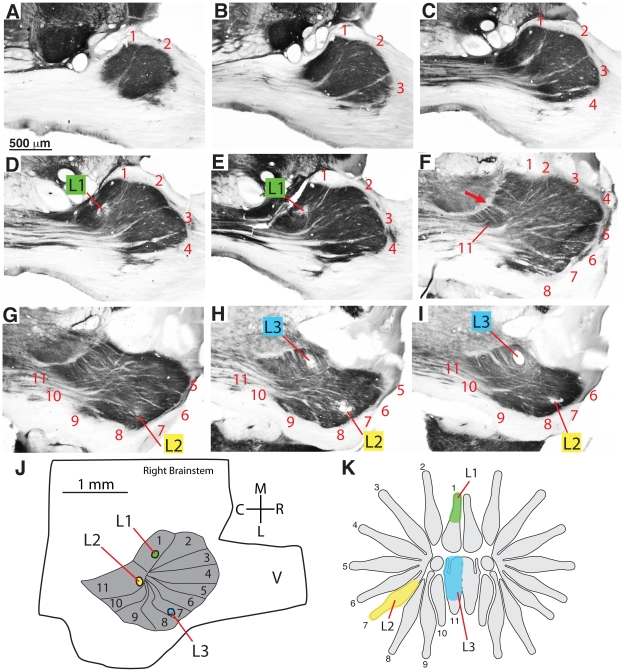
Microelectrode recordings from PrV related to brainstem anatomy. A–I. A series of sections through the brainstem showing the representation of the star in PrV and the locations of 3 microlesions made during the recordings (L1, L2, L3). J. Drawing of the brainstem showing the star subnucleus in PrV and the locations of the microlesions made during recordings. K. Receptive fields for neurons responding at lesioned sites L1, L2, and L3.

A favorable cutting angle (see materials and methods) revealed the entire set of 11 PrV modules in single sections of tissue. Figure 3 shows PrV and a rotated half of the star aligned to demonstrate how the PrV stripes correspond to the rays. This correspondence was determined by recording from PrV while stimulating the star with hand-held probes and calibrated von Frey hairs. Receptive fields were recorded and selected penetrations were marked with microlesions in 8 moles (see Figures 4 and 5 for two cases). The consistent finding was that ray number one was located rostro-medially in PrV (and at the deepest levels in the brainstem), followed by rays 2–8 (at shallower depths) as the electrode progressed laterally and caudally, and then rays 9–11 as the electrode was moved further caudally and back medially again. Details of this representation matched the topography of the star – for example rays 1 and 11 were aligned with one another on the star and in the brainstem representation (Figure 3). The differences in depth for the different ray representations corresponded to the angled orientation of the entire PrV nucleus in the brainstem. During the recordings, there was an obvious difference in the character of the responses from nasal rays, between penetrations in the trigeminal nerve and Sp5O, compared to PrV. The latter responded with greater magnitude and crisp, distinct single units compared to the former.

The above results indicated that PrV contains a large, histologically visible representation the star. However PrV in mammals receives input from mechanoreceptors distributed across the cranium and represents more than the nose. Therefore, the histologically distinct representation of the nasal rays must be a subnucleus of PrV – likely taking up a much greater area than remaining PrV cranial representations. Presumably, the representations of whiskers, oral structures, and other cranial mechanoreceptors in PrV are located adjacent to the chemoarchitectonically distinct star subnucleus. We obtained responses from the whiskers, tongue and oral structures just caudal to the star subnucleus, however more data are required to distinguish PrV representations of these structures from the mixture of traversing trigeminal tract fibers and potential responses from Sp5O. No responses from other cranial structures were identified within the star subnucleus.

### Sizes of Ray Representations in the Star Subnucleus of PrV

**Figure 6:**
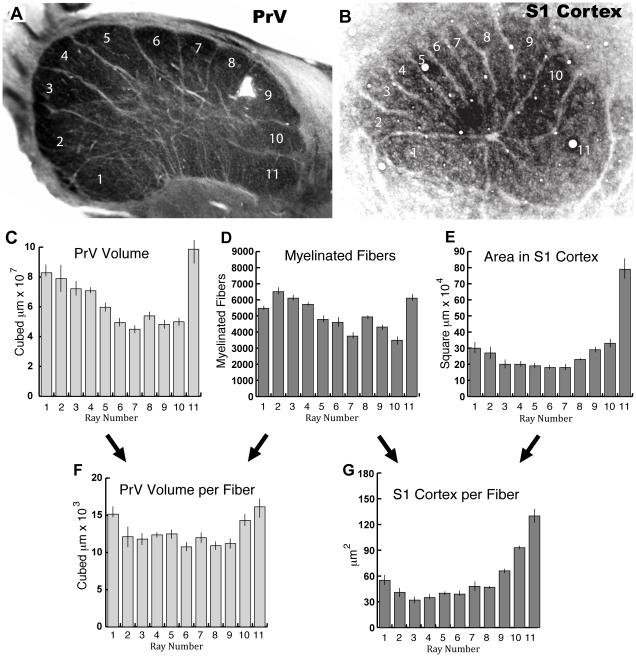
Comparison of myelinated fiber counts, PrV ray volumes, and cortical ray areas. A–B. A section of the principal nucleus (PrV) containing the star representation compared to a flattened section of cortex showing the primary somatosensory representation of the star (S1 Cortex), both processed for cytochrome oxidase. The areas of the ray representations in “A” are similar to the total PrV volumes of the ray representations from reconstructions of serial sections. Thus these images illustrate the general finding that ray 11, the tactile fovea, is more greatly over-represented at the level of the cortex (B) than in the brainstem (A). C. The mean PrV volumes for each ray representation (1–11) from the 4 reconstructed cases. D. Myelinated fiber counts for the 11 rays of 4 moles from a previous study [17]. E. Areas of cortex representing the rays of 4 moles from a previous study [17]. F. The mean volume of each ray representation in PrV per fiber (ratio of C to D). G. The mean S1 cortex per fiber for each ray representation (ratio of E to D). Note that D, E, and G (darker histograms) are from a previous investigation in 4 moles (adapted from[17]), whereas C and F are from the present study in 4 different moles. Bars in C–G are SEM.

All serial sections of 4 different PrV star subnuclei were photographed, aligned, and reconstructed (see materials and methods) to obtain the volume of each ray representation. The mean total volume of the star subnucleus from the 4 cases was 7.1 mm^3^. The mean volume of each ray representation is shown in Figure 6C. Ray 11, the tactile fovea, had the largest representation in the star subnucleus. The other ray representations were variable in size, with rays 1–5 generally having larger representations than 6–10. The overall pattern for the mean volumes of each ray representation in PrV was similar to the pattern observed in the single section in Figure 6A. Comparison of this visible PrV pattern to the representation visible in S1 (Figure 6B) suggests ray 11 is much more greatly magnified in the neocortex than in the brainstem.

### Comparison of Brainstem Volumes to Afferent Number and S1 Cortical Area

To investigate the relationship between brainstem representations and the neocortex in detail, we compared the results of the present study to previous results examining S1 cortex in star-nosed moles [17]. The previous investigation also included counts of all myelinated fibers (over 200,000 total) from the half star of each mole for which S1 cortex was examined (data in figure 6). These comparisons suggest some obvious conclusions about magnification of star representations at different CNS stations. First, the volumes of the ray representations at the level of the brainstem appeared different from the areas of cortex that represent the rays in S1 (Figure 6 C, E). Although ray 11 had the largest representation in both the brainstem and the neocortex, it took up roughly 25% of the star representation in S1, but only 14% percent of the PrV star subnucleus. Second, there was an overall similarity between the volume of each ray representation in PrV (Figure 6C) and the number of afferents from each corresponding ray (Figure 6D). The correlation coefficient (r) between fibers and trigeminal volume was 0.84 (p = 0.001). In contrast, there was little similarity between the number of fibers from each ray and the areas of each cortical representation (r = 0.293, p = 0.380), and r dropped to 0.12 when the outlying, 11th ray was removed from the analysis. Examining the ratio of trigeminal volume to afferent number (for each ray – Figure 6 F) and cortical representational area to afferent number (for each ray – Figure 6 G) further emphasized these relationships. The average trigeminal volume per afferent fiber for each ray appeared quite similar, with the exception of rays 1, 10, and 11 (Figure 6F). An ANOVA [F (10, 3) = 6.50 p<.0001] and Tukey's HSD test revealed that ray 11′s representation was significantly different (p<0.05) in this respect from all other rays except for 1 and 10. However this difference was slight compared to the relatively larger ratio of S1 cortex per afferent for ray 11 compared to other rays (Figure 6G, Catania and Kaas, 1997). Finally, the correlation between trigeminal volumes and S1 cortical area for each ray was significant (r = 0.66, p = .0276). The latter correlation seemed driven primarily by the expanded cortical representation of ray 11, and indeed r dropped to 0.167, p = 0.643 when this data point was removed. These results suggest that trigeminal and cortical levels of the CNS allocate territory to afferents differently, with PrV in the brainstem “over-representing” important afferents to some extent, but not nearly to the degree observed at the level of the neocortex.

## Discussion

The disproportionate representation of behaviorally important sensory receptors is a feature that has long been observed in somatosensory [28]–[29] visual [29], and auditory systems [30]. But the quantitative relationship between afferents from more important versus less important sensory inputs has been difficult to measure in most species. Star-nosed moles provide a favorable system for these measurements because the cortical areas representing the star are clearly visible [15] and different parts of the star have very different roles in behavior [14]. The central, 11^th^ pair of rays forms a tactile fovea that is preferentially used to explore objects and prey items, whereas the more peripheral rays 1–10 are used to guide saccadic movements of the star [14]. A previous investigation [17] of the relationship between afferent numbers from each ray and the size of corresponding cortical representations revealed a disproportionate representation of afferents from the tactile fovea, unlike the situation for mouse whiskers [5], but much like the disproportionate representation of the fovea reported in primates [13]. This raised the question of where the disproportionate representation of tactile foveal afferents first emerges in the mole's central nervous system. We addressed this question by examining the representation of the star in the mole's principal trigeminal nucleus (PrV) – the first station in the central pathway to the neocortex [18], [22].

### The Brainstem Trigeminal Nuclei in Star-Nosed Moles

Although the focus of the present investigation was on the star representation in PrV, we revealed interesting features of the star-nosed mole's entire trigeminal system. The most obvious finding was the greatly enlarged size of PrV compared to other species (Figure 1C, D). A similar result has been observed in other mammals with extreme sensory specializations [31] and in birds that rely heavily on tactile information while feeding [32]. In the case of star-nosed moles, PrV appears particularly large relative to the spinal trigeminal nuclei. For example, the hairy-tailed mole (Figure 1 A, B) has a comparatively small PrV (as in laboratory rodents) yet its spinal trigeminal nuclei are similar to those of the star-nosed mole. If PrV has indeed been selectively enlarged relative to spinal nuclei in star-nosed moles, this may shed important light on the differential functions of trigeminal nuclei. For example, it might be possible to correlate the relative size of different nuclei in different species to the proportions of different mechanoreceptors and nociceptors that have been correspondingly increased in specialized skin surfaces. But conclusions regarding the relative expansion of PrV in moles remain tentative because few studies (including our own) have quantified the total area of the spinal trigeminal nuclei, mainly because Sp5C projects far caudally and is not always included in brainstem sections [31]. It is possible that full reconstruction of the spinal trigeminal nuclei in star-nosed moles will reveal that all nuclei are expanded in star-nosed moles compared to most other small mammals.

Nevertheless, some conclusions about the absolute size of PrV in star-nosed moles can be made from the present analysis. For example, the total area of the PrV star representation was a mean of 0.71 mm^3^. The total volume of PrV reported for mice and rats is 0.37 mm^3^ and 0.56 mm^3^ respectively [31]. Thus the star representation in PrV alone is larger than the entire PrV of rats and mice. Because PrV in star-nosed moles must also represent additional cranial structures caudal to the star subnucleus, in areas that we have not fully identified (see results), the entire PrV of star-nosed moles is likely to be considerably greater than 0.71 mm^3^.

The results described above suggest an impressive overall expansion of PrV in star-nosed moles compared to PrV in other similarly sized small mammals. This specialization is even more extreme when considered at the level of sensory representations. For example, there is very little representation of the glabrous skin of the snout to be found in PrV of rodents [33]. Based on cortical maps [34], a similar situation is likely for snout skin in PrV of shrews, the sister group to the moles. Yet the representation of the glabrous snout in PrV of the star-nosed mole has expanded to the point of obscuring other parts of the cranial map and forming a chemoarchitectonically distinct subnucleus. This is a testament to the evolutionary flexibility of nervous systems in accommodating drastic modifications of the sensory periphery.

### Afferent Counts, PrV Ray Volumes, and S1 Cortex

A major goal of the present investigation was to determine how PrV represents the nasal rays of the star and to compare this result to ray representations in S1 cortex [17]. Our findings indicate that the volumes of ray representations in PrV are much more closely correlated with the afferent counts from each ray than was the case for the cortical representations of rays in S1 described in a previous study. Nevertheless, the afferents from the tactile fovea (ray 11) were over-represented in PrV. Thus there was not a direct, proportional representation of afferent number in PrV (as has been reported for rodents [25]). But the amount of this preferential representation of the fovea was far less at the brainstem level than found at the cortical level. Ray 11 receives 11% of the afferent fibers to the star, is represented by 14% of the star subnucleus in PrV, and takes up 25% of the star representation in S1 cortex. Together these findings suggest that PrV provides the first step in preferentially allocating CNS territory to behaviorally important afferents, but additional steps, perhaps including the thalamus, lead to the larger proportions observed in S1. This raises additional questions for further investigation. For example, how are the rays represented in the ventral posterior nucleus of the thalamus? A number of investigations in rodents have identified a whisker-related pattern (barreloids) in the thalamus of laboratory rats and mice [35]–[38] and this suggests the star representation might also be visible in appropriately sectioned and processed tissue from the thalamus. In addition, modular representations of the whiskers (barrelettes) have also been observed in the Sp5I and Sp5C of rodents [22], [24] and it seems likely that visible ray representations may be found in these nuclei of star-nosed moles as well. We have seen variably apparent modules in these nuclei in star-nosed moles that may represent the nasal rays.

But perhaps the most interesting question is what developmental mechanisms might result in the disproportionate allocation of CNS space to the most important afferents in star-nosed moles? Because the trigeminal representations develop before thalamus, and thalamic representations develop before cortex, there is potential for each station in the pathway to influence the next – at the earliest stages in map formation. A previous investigation of the star's embryonic and post-natal development revealed that ray 11 develops earliest [39]. It is the largest ray during much of embryonic development and the nerve endings that form sensory organs in the skin mature on ray 11 first. Thus one possibility is that ray 11 gets a head start in a competition for CNS space that is repeated multiple times (in the brainstem, thalamus, and then cortex) during development. This might be investigated by examining development at each station [40]–[42].
